# Hypermethylation of multiple genes as clonal markers in multicentric hepatocellular carcinoma

**DOI:** 10.1038/sj.bjc.6604016

**Published:** 2007-10-30

**Authors:** S Nomoto, T Kinoshita, K Kato, S Otani, H Kasuya, S Takeda, N Kanazumi, H Sugimoto, A Nakao

**Affiliations:** 1 Department of Surgery II, Nagoya University School of Medicine, 65 Tsurumai-cho, Showa-ku, Nagoya 466-8550, Japan

**Keywords:** hepatocellular carcinoma, multicentric occurrence, promoter hypermethylation, clonality, real-time methylation specific PCR

## Abstract

Hepatocellular carcinoma (HCC) is highly malignant and prone to multicentric occurrence. Differentiation between a true relapse of HCC and a second primary tumour appearing is of clinical importance. At this point, no convenient method is available to determine the origin of these HCCs. Tissue samples were obtained from 19 patients and analysed for the promoter hypermethylation status of multiple tumour suppressor genes (*p16, DAP-Kinase, MGMT, GSTP1, APC, RIZ1, SFRP1, SFRP2, SFRP5, RUNX3*, and *SOCS1*) using methylation-specific PCR (MSP). Methylation status was used to determine tumour clonality. In each of the 19 cases, at least one tumour was recognised as having an aberrantly methylated gene. The frequency of the methylation in tumour tissue was 57.1% in *p16*, 2.4% in *DAP-kinase*, 23.8% in *GSTP1*, 90.5% in *APC*, 45.2% in *RIZ1*, 64.3% in *SFRP1*, 59.5% in *SFRP2*, 28.6% in *SFRP5*, 47.6% in *RUNX3*, and 54.8% in *SOCS1*, while in *MGMT*, no aberrant methylation was detected. The methylation status of these genes was assessed using MSP as being either positive or negative, and was used to determine the tumour clonality. The clonality of every tumour could be decided even with lesions that could not be judged by clinical diagnosis or by another molecular method (mt DNA mutation). Determining the methylation status of multiple genes in multicentric HCC was useful as a clonal marker and provided useful information for characterising the tumour. From our findings, multicentric HCCs tend to occur more independently than metastatically from the original tumour. Expanded study should be pursued further for a better understanding of the molecular mechanism of hepatocarcinogenesis.

One of the most frequent tumour types worldwide is hepatocellular carcinoma (HCC). It is most commonly associated with chronic hepatitis B and C virus infections, with chronic exposure to the mycotoxin, aflatoxin B1 (AFB1), and is a complication of alcoholic cirrhosis. Death is often due to liver failure associated with cirrhosis and/or rapid outgrowth of multilobular HCC (Feitelson *et al*, 2002). Although early HCC may be cured by surgical resection, the central issue of this fatal disease is that it is prone to multicentric occurrence. The progression and outcome of truly relapsed HCC are distinct compared to a second primary tumour, and thus clonal analysis of initial and recurrent HCC is of clinical significance. Although several studies have addressed HCC clonal determination (Yamamoto *et al*, 1999; Chen *et al*, 2000; Ochiai *et al*, 2000; Nomoto *et al*, 2002), the reported molecular methods are still of limited clinical use or are technically quite challenging.

The development of HCC is a multistep process associated with changes in host gene expression, some of which correlate with the appearance and progression of tumour. Loss of heterozygosity and several types of mutations in tumour suppressor genes are reported to be involved in cancer development (Feitelson *et al*, 2002).

Recently, epigenetic silencing is also considered to be a significant contributor to human carcinogenesis. Silencing of tumour suppressor genes by methylation of the CpG-rich promoter region has been reported in many kinds of human cancers. Some tumour suppressor genes such as *p16*, VHL, and MLH1 have been found to harbour promoter hypermethylation associated with loss of protein expression in cancer cells (Herman *et al*, 1994, 1998; Merlo *et al*, 1995). Several tumour types have also shown aberrant methylation at the CpG island in other genes, including the FAP-associated gene *APC* (Esteller *et al*, 2000), detoxifying gene *GSTP1* (Lee *et al*, 1994), DNA repair gene *MGMT* (Esteller *et al*, 1999a), the potential metastasis inhibitor gene DAP-kinase (Esteller *et al*, 1999b), and Rb interact gene *RIZ1* (Du *et al*, 2001). Members of the frizzled-related protein family (SFRP-1, -2, and -5), receptors for Wnt family members, were also reported to show promoter hypermethylation in colorectal cancers (Suzuki *et al*, 2002). We have reported previously hypermethylation of RUNX3 gene, locus at 1p36, where chromosomal deletion has frequently been found in various types of cancers, including HCC (Mori *et al*, 2005). Aberrant methylation was found in 65% of primary HCC tumour samples in *SOCS1* gene (Yoshikawa *et al*, 2001).

In the present study, we analysed the promoter hypermethylation status of the human *p16, MGMT, GSTP1, DAP-kinase, APC, RIZ1, SFRP1, SFRP2, SFRP5, RUNX3*, and *SOCS1* genes in the multiple tumour DNA and the corresponding normal DNA of HCC, and tried to apply it to determine their clonality. The clonality of all of the multiple lesions could be identified by this combined analysis to determine the promoter hypermethylation status of multiple tumour suppressor genes.

## MATERIALS AND METHODS

### Clinical HCC samples

Simple nodular HCC and adjacent nontumourous liver tissues from 19 patients, which were used in this study, were surgically resected in Nagoya University Hospital between 1989 and 2000. Thirteen of the 19 patients had synchronous multiple HCC, which were resected during their first operation. In the other six patients, hepatic tumours reappeared between 10 months and 5 years after the first curative operation, and they were resected by a surgical approach. Serologic markers for hepatitis B and C virus infections and *α*-fetoprotein levels were recorded.

### DNA extraction

All specimens were immediately fresh-frozen after the resection and stored at −80°C. Serial 10 *μ*m sections were cut with a microtome. After staining with haematoxylin and eosin for the histologic examination, the other sections were used for DNA extraction. With the exception of a small part comprised of fatty and necrotic cells, nearly all (>80%) of our sections were cancerous in nature. Normal and tumour DNA were prepared as described previously (Goelz *et al*, 1985).

### Methylation-specific PCR

Sodium bisulphite treatment converts nonmethylated cytosine residues to uracil, while methylated cytosine within CpG islands remains unaffected. Briefly, 2 *μ*g of DNA was denatured by NaOH and modified by sodium bisulphate. DNA samples were then purified using the Wizard purification resin (Promega Corp., Madison, WI, USA), treated again with NaOH, precipitated with ethanol, and resuspended in water. The primers and PCR conditions used in this study are summarised in Table 1. Nonmethylated promoter-specific PCR was always performed as well for each gene.

Amplifications were carried out in 96-well plates. All samples were run in triplicate and repeated in quadruple if any difference was noted. Each plate included multiple water blanks for a negative control. Lymphocyte DNA from a healthy individual was used as the negative control for 11 of the genes. The same lymphocyte DNA was methylated *in vitro* with excess Sss I Methyltransferase (New England Biolabs Inc., Beverly, MA, USA) to generate completely methylated DNA at all CpG and used as the positive control. Each PCR product was loaded directly onto 2.5% agarose gels, stained with ethidium bromide, and visualised under ultraviolet illumination.

### Determining tumour clonalities

Tumour clonality was determined by the methylation status of multiple tumour suppressor genes. Basically, when the methylation status of the genes was the same as the original tumour, we assumed that the tumour was a metastatic lesion. Because of the loss of progression advantage of tumorigenesis, and if there were a loss of promoter methylation at least in the second or third lesion in a gene, those tumours were determined as independent. Thus, we supposed that a metastatic tumour could gain promoter hypermethylation in a gene that was not methylated in the first tumour. The clonality determined by the methylation status of the genes was compared with clinical diagnosis or the decision according to the mutation status in the mitochondrial genome (Nomoto *et al*, 2002).

## RESULTS

### Promoter hypermethylation of tumour suppressor genes in HCC tissue

We checked the status of promoter hypermethylation for 11 genes that were reported to show frequent promoter hypermethylation in HCC or other types of cancer tissues by MSP. When an appropriate band was seen on a gel by loading the product of MSP, the methylation status was considered to be positive. The frequency of methylation in tumour tissue was as follows: 57.1% (24 of 42) in *p16*, 0% (0 of 42) in *MGMT*, 28.6% (12 of 42) in *GSTP1*, 78.6% (33 of 42) in *APC*, 2.4% (1 of 42) in DAPK, 45.2% (20 of 42) in *RIZ1*, 64.3% (27 of 42) in *SFRP1*, 59.5% (25 of 42) in *SFRP2*, 28.6% (12 of 42) in *SFRP5*, 47.6% (20 of 42) in *RUNX3*, and 54.8% (23 of 42) in *SOCS1*. In noncancerous tissue, on the other hand, hypermethylation was found with the following frequency: 68.4% (13 of 19) in *APC*, 10.5% (2 of 19) in *DAPK*, 5.2% (1 of 19) in *RIZ1*, 47.4% (9 of 19) in *SFRP1*, 52.6% (10 of 19) in *SFRP2*, 10.5% (2 of 19) in *SFRP5*, 10.5% (2 of 19) in *RUNX3*, and 2.5% (1 of 19) in *SOCS1*. In addition, we checked the methylation status in 32 tumours with corresponding normal tissues from patients with single HCC. The methylated DNAs were detected with the following frequency: 65.6% (21 of 32) in p16, 3.1% (1 of 32) in *MGMT*, 31.3% (10 of 32) in *GSTP1*, 87.5% (28 of 32) in *APC*, 0% (0 of 32) in DAPK, 56.3% (18 of 32) in *RIZ1*, 56.3% (18 of 32) in *SFRP1*, 53.1% (17 of 32) in *SFRP2*, 28.1% (9 of 32) in *SFRP5*, 46.9% (15 of 32) in *RUNX3*, and 46.9% (15 of 32) in *SOCS1*. There were no significant statistical differences between cases of multiple HCC and single HCC. All cases examined in this study had methylation in at least one gene. The frequency of methylated cases in the studied genes is summarised in Table 2. The information on the patients and tumours is summarised in Table 3.

### Clonality by methylation or nonmethylation

**Figure 1:**
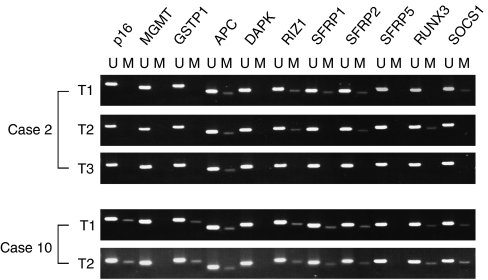
Representative cases of methylation status in multiple genes provided by methylation-specific PCR in tumour and corresponding normal tissue in patients with multicentric HCC. The different pattern of methylated genes in each tumour shows an independent occurrence in case 2. When the aberrant methylation in the same genes was shown in both tumours in case 10, the judgement was metastases.

**Figure 2:**
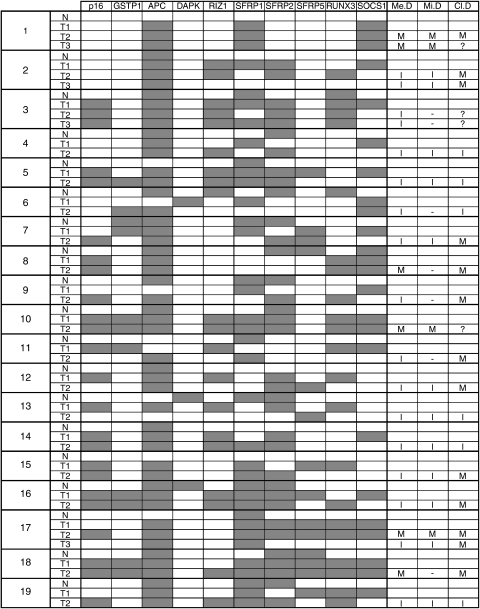
Summary of methylation of *p16, GSTP1, APC, DAPK, RIZ1, SFRP1, SFRP2, SFRP5, RUNX3*, and *SOCS1* in multiple HCC and corresponding normal tissues. Filled boxes indicate methylated loci. Open boxes indicate nonmethylated promoter loci. M, metastasis; I, independent lesion; ?, indeterminate. The largest tumour in the first operation was listed as the first primary lesion. Clonalities of mtDNA (Mi.D) were shown in a previous article (Nomoto *et al*, 2002). Clinical diagnosis (Cl.D) was determined based on the Classification of Primary Liver Cancer in Japan. The results decided according to methylation status are shown in the column of Me.D. N, corresponding normal tissue; T1, original tumour; T2, second tumour; T3, third tumour.

In all of the cases examined in this study, clonality was determined by the methylation status of *p16, GSTP1, APC, RIZ1, SFRP1, SFRP2, SFRP5, RUNX3*, and *SOCS1*. *MGMT* and *DAPK* were not useful because of the low frequency of methylated cases. Basically, we judged that clonality had a different origin if the methylation status was different from the original tumour in at least one gene. In particular, if a second (or third) tumour had a loss of methylation in the same gene, it was considered an independent tumour. For example, in case 2, T2 had a loss of methylation in *SOCS1* and *SFRP1*, and T3 had lost methylation additionally in *RIZ1* and *RUNX3* genes. It was therefore assumed that the tumours had a separate origin (Figure 1). Further, we supposed that a metastatic second (or third) tumour could have methylation in a gene even if the original tumour did not. For instance, the T2 tumour of case 17 was methylated in *p16* gene, although the first tumour did not have methylation in the gene. Clonality was determined by comparing the methylation status, with the result being decided by the mutation in the mitochondrial genome. In cases where clonality could be determined by mitochondrial mutation, all of the results were consistent with the clonality decided by methylation status. In other cases, we could judge the clonalities in all tumours. Then, only 6 of 23 (26.1%) tumours were determined to be metastases from the original tumour, although 68.4% (13 of 19, excluding four undetermined cases) were supposed to be metastatic tumours from the clinical diagnoses. These results are summarised in Figure 2.

## DISCUSSION

Hypermethylation of normally nonmethylated CpG islands in the promoter regions often occurs in important tumour suppressor genes such as VHL, hMLH1, and *p16* (Herman *et al*, 1994, 1998; Merlo *et al*, 1995). In HCC tissues, frequent aberrant methylation in *p16* gene was reported (Wong *et al*, 1999). Recently, loss of expression of other interesting genes, the detoxifying gene *GSTP1* (Zhong *et al*, 2002) and Rb interact gene *RIZ1* located within 1p36.13–p36.23, has been found in HCCs through promoter methylation (Du *et al*, 2001). The DNA repair gene, *MGMT*, frequently inactivated in brain, colorectal, lung, and lymphomas (Esteller *et al*, 1999a), and the potential metastasis inhibitor DAP-kinase gene altered in lymphomas, leukaemias, and lung cancer, were also reported with aberrant methylation (Katzenellenbogen *et al*, 1999; Esteller *et al*, 1999b). We reported frequent methylation in *SOCS1* and another gene mapped at 1p36, *RUNX3*, in HCC tissues (Okochi *et al*, 2003; Mori *et al*, 2005). In addition, one of the major player genes involved in cancer development in the Wnt pathway, *APC*, was also reported to show methylation-dependent silencing of expression (Esteller *et al*, 2000). Other Wnt signal genes in the SFRP family have been reported to have aberrant methylation in colon cancers (Suzuki *et al*, 2002). To our knowledge, ours is the first report regarding promoter hypermethylation in SFRP genes with HCC patients, and frequent methylation was confirmed in *p16, GSTP1, RIZ1, SOCS1*, and *RUNX3*. In addition, SFRP-1, -2, and -5 genes were found frequently methylated in HCC tissue. In a DNA repair gene *MGMT*, no methylation was detected. We could detect very few instances of aberrant methylation in *DAP-kinase* because most of the tumours were independent lesions, not metastases. Interestingly, *APC* gene was commonly methylated in fully normal liver tissues (88.2%, 15 of 17), and reduced methylation frequency was detected in cirrhotic liver and/or tissue in chronic hepatitis (21.6%, 11 of 51). After tumour development, the methylation status again became high (82.4%, 61 of 74). This might be related to a potential for proliferation, and *APC* might have an important role in the regulation of regeneration. Loss of *APC* methylation in cirrhotic and inflammatory cases could possibly be due to infiltration of fibroblasts and/or inflammatory cells. In *SFRP1* and SFRP2 genes, although very little aberrant methylation was shown in fully normal liver, frequent methylation was found in corresponding noncancerous tissues. Methylation in *SFRP-1* and *SFPR-2* genes might be related in premalignant situations or very early events in hepato-carcinogenesis.

Several studies are relevant regarding the clonality decision for multiple HCC. When judging by genetic mutations in p53 gene or mitochondrial genome (Oda *et al*, 1992; Nomoto *et al*, 2002), or by integrating the genomic pattern of hepatitis B virus (Yamamoto *et al*, 1999), the specificity of the decided clonalities was accurate. Considering sensitivity, the clonality decision was limited only to cases with mutations of the genes or tumours based on hepatitis B virus. We decided the clonality in all multicentric tumours using MSP in multiple tumour suppressor genes. It is worth mentioning that our method was successful in all cases, including cases 1 and 10, which could not be determined clinically, in cases 12, 16, and 17, where clinical diagnosis was clearly wrong (as confirmed by mitochondrial status), and even in cases 11 and 18, when mtDNA testing was noninformative. Regarding the sensitivity, this method is superior to both standard clinical acumen and all other molecular methods.

The specificity of our study is considered reliable because the results of the judgement were all consistent with the decisions that could be determined by mutations in the mitochondrial genome. Although the promoter hypermethylation event in tumour cells might be reversible, examining more than 10 genes improves the accuracy of the study.

Had we increased the number of tumour suppressor genes to be checked, which were frequently methylated in HCC tissue, it would have been more advantageous not only for determining clonality but also for assessing the malignant potential of the tumours. Most of the tumours are associated with a multistep process of development with changes in host gene expression (Feitelson *et al*, 2002). It could be very important to know the altered multiple pathways in a tumour for appropriate therapy in future.

Regarding the heterogeneity of HCC in a tumour, the mitochondrial mutation and the methylation status of 11 genes for DNAs obtained from five nonfatty parts of a tumour were checked, and the same mitochondrial mutation and similar methylation pattern were found in each DNA (data not shown). This might occur rarely in multiple genomic changes in a simple nodular HCC like the tumours examined in this study.

Surprisingly, most of the relapsed tumours diagnosed as metastases by clinical and pathological findings were determined to be independent lesions. These findings imply that any region of the liver where HCCs occurred has a potential for recurrence, because most of the HCCs are considered to grow on the basis of cirrhotic liver or chronic hepatitis. After further research on the liver stem cells and reducing the risks of operation, transplantation might take the place of resection in the future. Currently, we have to confirm the clonality of multiple HCCs for the future direction of therapy.

We have clearly shown that the methylation status of multiple tumour suppressor genes in multicentric HCC is useful as a clonal marker. By increasing the number of cases and frequently methylated genes related to tumour initiation and progression, the clonality would be shown more certainly. Furthermore, studies on checking methylation and searching mutations in multiple tumour suppressor genes should be expanded to screen normal tissue obtained by biopsy from patients with cirrhotic liver or chronic hepatitis to determine which gene(s) are most related to tumour initiation. This would be an opportunity to prevent the development of HCC. A greater understanding of the molecular pathogenesis of HCC may yield new markers for tumour staging, for assessment of the relative risk of tumour formation, and provide new opportunities for therapeutic intervention.

## Figures and Tables

**Table 1:** Summary of primer sequences and PCR product size

**Gene**	**Forward 5′–3′**	**Reverse 5′–3′**	**Size**
*p16*	TTATTAGAGGGTGGGGCGGATCGC	GACCCCGAACCGCGACCGTAA	150
*MGMT*	CGAATATACTAAAACAACCCGCG	GTATTTTTTCGGGAGCGAGGC	122
*GSTP1*	AGTTGCGCGGCGATTTC	GCCCCAATACTAAATCACGACG	140
*APC*	GAACCAAAACGCTCCCCAT	TTATATGTCGGTTACGTGCGTTTATAT	74
*DAPK1*	GGATAGTCGGATCGAGTTAACGTC	CCCTCCCAAACGCCGA	101
*RIZ1*	GGATTCGCGGTGATTTACGA	CTACGAAACTAAAAAACTCCGAAAC C	121
*SFRP1*	CGTTCGCGAGGGAGGCGATT	AACCGCCCCGCGCAACCAAT	97
*SFRP2*	TCGCGGGTCGGGTAAATAAGT	GCTACCCGACTTACCGCCAA	103
*SFRP5*	TTAGTCGGGGCGTTCGTAGC	CTCGATACCCGACGACCCAA	126
*RUNX3*	CGTCGGGTTAGCGAGGTTTC	GCCGCTACCGCGAAAAACGA	120
*SOCS1*	CGCGCGGGGTTTTCGTAGTA	CTAACTCCAACCGTCCGACC	130

**Table 2:** Summary of results of aberrant methylation

**Gene**	**Tumours in multiple HCC case (*n*=42)**	**Normals in multiple HCC case (*n*=19)**	**Tumours in single HCC case (*n*=32)**	**Normals in single HCC case (*n*=32)**	**Normal liver (*n*=17)**
*p16*	24 (57.1%)	0 (0%)	21 (65.5%)	0 (0%)	0 (0%)
*MGMT*	0 (0%)	0 (0%)	1 (3.1%)	0 (0%)	0 (0%)
*GSTP1*	12 (28.6%)	1 (5.2%)	10 (31.3%)	1 (3.2%)	0 (0%)
*APC*	33 (78.6%)	13 (68.4%)	28 (87.5%)	22 (68.8%)	15 (88.2%)
*DAPK1*	1 (2.4%)	2 (10.5%)	0 (0%)	0 (0%)	0 (0%)
*RIZ1*	20 (45.2%)	1 (5.2%)	18 (56.3%)	0 (0%)	1 (5.9%)
*SFRP1*	27 (64.3%)	9 (47.4%)	18 (56.3%)	14 (43.8%)	1 (5.9%)
*SFRP2*	25 (59.5%)	10 (52.6%)	17 (53.1%)	17 (53.1%)	2 (11.8%)
*SFRP5*	12 (28.6%)	2 (10.5%)	9 (28.1%)	3 (9.4%)	1 (5.9%)
*RUNX3*	20 (47.6%)	2 (10.5%)	15 (46.9%)	2 (6.3%)	1 (5.9%)
*SOCS1*	23 (54.8%)	1 (5.2%)	15 (46.9%)	1 (3.2%)	0 (0%)

Abbreviation: HCC=hepatocellular carcinoma.

**Table 3:** Information of the patients and tumours

**Case**	**Sex**	**Age (year)**	**Lag (mo)**	**HBs-Ag/HCV-Ab**	**Cirrhosis**	**Size (cm)**	**Diff**
1	M	63		−/+	+	0.8 × 0.8 × 0.6	Mod
						0.6 × 0.6 × 0.5	Mod
			38			2 × 1.7 × 1.3	Mod
							
2	M	71		−/−	+	11 × 9 × 8	Mod
						2 × 1.8 × 1.6	Mod
			10			2.9 × 2.2 × 1.8	Mod
							
3	M	59		−/+	+	3 × 3 × 3	Mod
			15			2.1 × 2 × 1.8	Mod
			15			0.9 × 0.9 × 0.9	Mod
							
4	F	57		+/−	+	4 × 3.5 × 3.5	Mod
			116			1 × 1 × 0.8	Mod
							
5	M	61		−/+	+	2.5 × 2.3 × 2.1	Mod
			47			3 × 2.6 × 2.3	Mod
							
6	M	36		+/−	+	3 × 3 × 2	Mod
			65			3 × 3 × 1	Mod
							
7	M	68		−/+	+	3 × 3 × 3	Mod
						2.7 × 2.7 × 2.5	Mod
							
8	M	54		−/−	−	10 × 9.5 × 8.5	Mod
						3 × 3 × 2	Mod
							
9	M	69		−/+	+	4 × 4 × 4	Mod
						1.5 × 1.5 × 1.5	Mod
							
10	M	47		+/−	+	8.8 × 8 × 7.5	Mod
						2 × 2 × 1.5	Mod
							
11	M	49		−/+	+	3 × 2.4 × 2.4	Mod
						3 × 2.2 × 2	Mod
							
12	M	56		−/−	−	5 × 5 × 4.7	Mod
						2.2 × 2 × 1.8	Mod
							
13	M	72		−/+	+	2.5 × 1.8 × 1.5	Mod
						2 × 1.6 × 1.2	Well
							
14	M	54		−/+	+	2 × 2 × 2	Mod
						0.7 × 0.6 × 0.6	Well
							
15	M	65		−/+	+	4.5 × 4 × 4	Mod
						4.5 × 3.5 × 3	Mod
							
16	F	63		−/+	+	3 × 2.4 × 2	Mod
						2 × 1.7 × 1.5	Mod
							
17	M	56		−/+	+	1.9 × 1.2 × 1.2	Mod
						1.4 × 1.2 × 1.2	Mod
						1.3 × 1.2 × 1.1	Mod
							
18	F	66		−/+	+	2.2 × 1.9 × 1.8	Mod
						1.6 × 1.4 × 1.4	Mod
							
19	M	60		−/+	+	3.6 × 3.4 × 3	Poor
						2.4 × 2.2 × 2	Mod

Lag means the time between the first and second operation (mo=month).
